# Application of Anchoring Technique in Unilateral Percutaneous Vertebroplasty for Neurologically Intact Kümmell's Disease

**DOI:** 10.1155/2020/4145096

**Published:** 2020-05-24

**Authors:** Rongqing Qin, Xing Zhang, Hongpeng Liu, Bing Zhou, Pin Zhou, Chuanliang Hu

**Affiliations:** ^1^Department of Spinal Surgery, Gaoyou Hospital Affiliated Soochow University, Gaoyou, Jiangsu 225600, China; ^2^Department of Orthopedics, Gaoyou People's Hospital, Gaoyou, Jiangsu 225600, China; ^3^Department of Orthopedics, Gaoyou Hospital of Integrated Traditional Chinese and Western Medicine, Gaoyou, Jiangsu 225600, China

## Abstract

**Purpose:**

We aimed to present our experience in anchoring technique and evaluate the efficacy and safety of unilateral percutaneous vertebroplasty in patients with neurologically intact Kümmell's disease.

**Methods:**

From January 2014 to December 2017, 29 patients (17 males and 12 females) with neurologically intact Kümmell's disease were operated on using anchoring technique in unilateral percutaneous vertebroplasty (PVP). Ages of the enrolled patients ranged from 67 to 81 years (mean 73.8 years). Clinical efficacy was evaluated by back pain visual analogue scale (BP-VAS) score, Oswestry disability index (ODI) score, as well as the height of anterior border and the kyphotic angle of the involved vertebral body on a standing lateral radiograph. The safety of PVP was assessed by surgical-related complications, including bone cement leakage and neurological deficit.

**Results:**

All 29 patients underwent the PVP procedure successfully. The mean operation time was 35 ± 12 min. And all patients were able to walk/ambulate with a thoracolumbar brace after 12 to 24 hours, staying in bed postoperatively. Significantly statistical differences were observed in both BP-VAS and ODI scores at each time point of follow-up when compared with the preoperative condition (*P* < 0.05). Besides, statistically significant improvement in radiographic measurements such as kyphotic angle and the height of the anterior border of the involved vertebral body between the preoperative and postoperative assessments was also observed (*P* < 0.05) and asymptomatic leakage of cement occurred in 7 of 29 cases (24.1%).

**Conclusions:**

We considered that the anchoring technique in unilateral PVP could provide an effective and safe alternative for neurologically intact Kümmell's disease.

## 1. Introduction

Kümmell's disease refers to delayed vertebral collapse, dynamic instability, and progressive kyphosis with prolonged back pain and/or neurological deficit after an asymptomatic or mild period [1, 2]. It was first described in 1895 by Dr. Kümmell's [3, 4]. Clinically, it occurs mostly in osteoporotic patients, and particularly in the thoracolumbar zone [5]. The ability to diagnose the intravertebral vacuum cleft as a sign of Kümmell's disease has improved with the development of imaging technology [6] and several terms have been used to describe this condition, including intravertebral vacuum cleft [7, 8], intravertebral vacuum sign [5], vertebral osteonecrosis [2], delayed vertebral collapse [9], and vertebral pseudarthrosis [10]. Most patients have no neurological symptoms. Patients with Kümmell's disease are refractory to conservative treatment such as narcotic analgesics and bed rest [11]. The goal of the surgical intervention is to ease the back pain and to prevent the involved vertebra from further collapse, and to avoid kyphotic deformity. For patients with neurologically intact Kümmell's disease, open surgery is not an advisable option, especially in elders or osteoporotic patients, and minimally invasive surgery such as percutaneous vertebroplasty (PVP) has acquired good clinical outcomes [12–14].

The objective of the present study was to present our experience in the anchoring technique and evaluate the efficacy and safety of PVP in Kümmell's disease without neurological deficit.

## 2. Materials and Methods

### 2.1. Patient Population

After the Institutional Review Board approval, 29 patients (17 males and 12 females) who underwent anchoring technique in PVP for neurologically intact osteoporotic Kümmell's disease from January 2014 to December 2017 were enrolled in this study. The age range was from 67 to 81 years (mean 73.8 years) at the perioperative period. The mean duration of clinical symptoms was 4.3 ± 1.2 months (range, 2–7 months). All patients were Kümmell's disease with single-segment lesion located at the following thoracic (T) or lumbar (L) vertebrae: T7 (2 cases), T10 (2 cases), T11 (4 cases), T12 (9 cases), L1 (7 cases), L2 (3 cases), and L3 (2 cases). 16 patients had a history of minor trauma such as carrying heavy objects, falling on foot, or having cycling bumps, and the remaining 13 patients had no obvious history of trauma. All patients experienced radiographs, computed tomography (CT), and magnetic resonance imaging (MRI). Osteoporosis was confirmed by dual-energy X-ray absorptiometry preoperatively. In addition, among the 29 patients, 8 patients had internal medical comorbidities such as diabetes and/or hypertension. Conventional preoperation examinations, pulmonary function tests, and echocardiography were performed in all patients. Baseline characteristics were shown in Table 1.

### 2.2. Inclusive and Exclusive Criteria

#### 2.2.1. Inclusive Criteria

(1) Patients had a history of minor trauma or no obvious history of trauma; (2) patients without neurological deficit; (3) patients with collapsed vertebra within a radiotranslucent zone on plain standing radiographs; (4) patients with CT showing the intravertebral cleft (or vacuum sign) with or without sclerotic margin; (5) patients with the presence of a low signal intensity in the location of the cleft on T1-weighted images, and a high or low signal intensity on T2-weighted MRI, which depends on whether it is a liquid or a gas in the cleft; (6) patients with osteoporosis; (7) patients with 1-year follow-up at least.

#### 2.2.2. Exclusive Criteria

(1) Patients with neurological deficit; (2) patients who had a history of thoracolumbar surgery; (3) patients with infected lesions on the puncture path; (4) patients with underlying malignancy; (5) patient with serious physical illnesses, abnormal blood coagulation function, or mental disorder; (6) patients who were unable to tolerate the PVP procedure under local anesthesia.

### 2.3. Surgical Procedures

**Figure 1:**
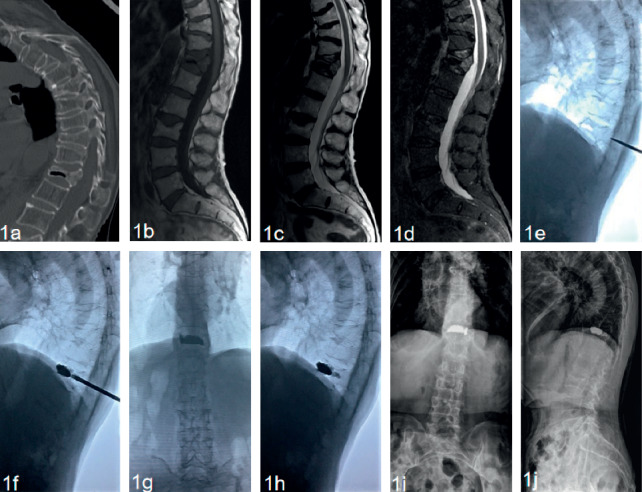
A 78-year-old male patient with Kümmell's disease at T12 treated by PVP. (a) Preoperative sagittal CT scan showed the intravertebral vacuum sign; (b) sagittal T1-weighted MR image showed a low signal intensity in the location of the cleft; (c, d) sagittal T2-weighted MR image and short tau inversion recovery (STIR) image showed a well-defined low signal intensity in the location of the cleft; (e) the height of the affected vertebral body was partly recovered at a hyperextension position; (f) the low-viscosity PMMA adhered to the previous high-viscosity mass and diffused into the intervertebral trabecular space; g-h: X-ray immediately after operation showed the bone cement filled the cleft without leakage. The symmetric distribution of the bone cement was verified (g), the low-viscosity PMMA was firmly fixed in the vertebral body like a ship anchor to prevent the slide of the highviscosity mass of bone cement (h); (i, j) no delayed displacement of bone cement, or adjacent vertebral fracture were observed at the last follow-up.

**Figure 2:**
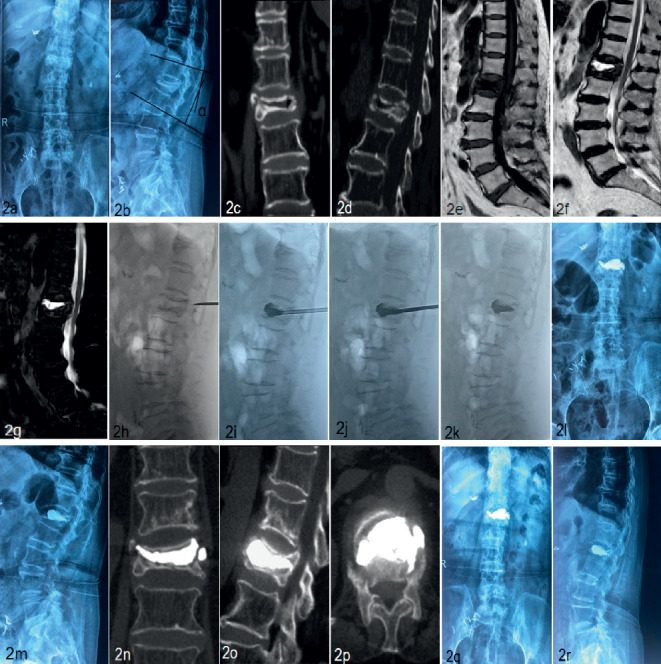
A 75-year-old female patient with Kümmell's disease at L1 treated by PVP. (a, b) Preoperative radiographical images, the kyphoticangle *α* was marked with black lines; (c, d) coronal and sagittal CTscans showed the intravertebral vacuum sign; (e) the sagittal T1-weighted MR image showed a low signal intensity in the location of the cleft; (f, g) the sagittal T2-weighted MR image and STIR image showed a welldefined high signal intensity in the location of the cleft; (h) the height of the L1 vertebral body was partly recovered at a hyperextension position; (i–k) first, high-viscosity PMMA was injected under fluoroscopy during the early stage of dough period to plug the gap (i). Then, low-viscosity PMMA was prepared at the early stage of the wire drawing period and injected cautiously (j); (l, m) X-ray immediately after operation showed the bone cement filled the cleft; (n–p) CT scans at three month after operation; (q, r) no delayed displacement of bone cement or adjacent vertebral fracture were observed at the last follow-up.

Patients were in a prone position with abdomen vacant. The PVP procedure was performed under stringent sterile conditions. After the localization of the affected vertebral body, local anesthesia was performed on the skin, fascia, and articular capsule before manipulative reduction. Both lower extremities were elevated and pulled backward by two assistants, while head side traction via the patient's axilla and shoulder was performed by another assistant, and the operator pressed the back of the patient when it was inconsistent with the involved vertebral body. Manipulative reduction was conducted within the patient's tolerance and under fluoroscopic guidance. Then, further silicone pads were placed on the chest and ilium to maintain a maximum hyperextension position. After successful positioning, the surface projection of one-side pedicle was marked as a skin puncture point. Then, the guide needle, dilation cannula, and work cannula were replaced successively under fluoroscopy. The ideal position of the top of the push rod was the anterior-middle 1/3 junction of the involved vertebra. Bone cement was modulated twice to complete the process of perfusion and anchorage in the vertebral body. First, high-viscosity polymethyl methacrylate (PMMA) was injected under fluoroscopy during the early stage of the dough period to plug the gap, and the resistance was not great because of the existing gap. Second, the bone cement was prepared at the early stage of the wire drawing period and injected cautiously and slowly. Under fluoroscopy, it appeared that the low-viscosity PMMA adhered to the previous bone cement mass and diffused into the intervertebral trabecular space. The injection was stopped until the diffusion was sufficient. The total dosage of the bone cement injected twice was approximately 4–7 ml. After the surgery, all patients were given antiosteoporotic treatment and rehabilitative exercise of the muscle strength of the waist and back Typical cases were shown in Figures 1 and 2.

### 2.4. Efficacy Evaluation

Clinical outcomes were evaluated by back pain visual analogue scale (BP-VAS) score from 1 to 10 (1, no pain; 10, worst possible pain) and Oswestry disability index (ODI) score before the operation and at 1 day, 1 month, 6 month, 12 months, and last follow-up postoperatively. Besides, the kyphotic angle and the height of anterior border of the involved vertebral body on a standing lateral radiograph were recorded at the above follow-up points. The kyphotic angle was marked with black lines in Figure 2(b). The safety of PVP was assessed by surgical-related complications, including bone cement leakage, neurological deficit, pulmonary embolism, thermal injury, infection, delayed displacement of bone cement, or adjacent vertebral fracture. The leakage of bone cement was detected using postoperative immediate CT scans. In addition, the time of operation, ambulation, and hospitalization was recorded.

### 2.5. Statistical Analysis

Statistical analysis was implemented by SAS software (version 9.4). Results were presented as mean ± standard deviation. The one-way ANOVA Dunnett *t*-test was used for assessing the significance of differences between preoperative and postoperative BP-VAS and ODI sores, respectively. *P* values of less than 0.05 were accepted for significance.

## 3. Results

**Figure 3:**
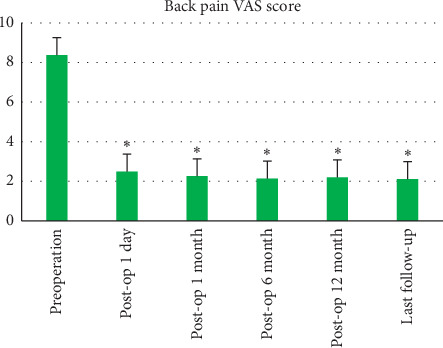
Histograms for back pain VAS scores.

**Figure 4:**
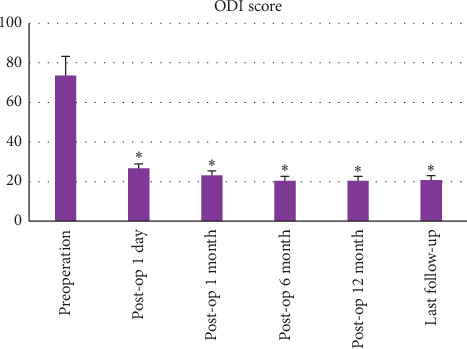
Histograms for ODI scores (*n* = 29, ^*∗*^*P* < 0.05).

**Figure 5:**
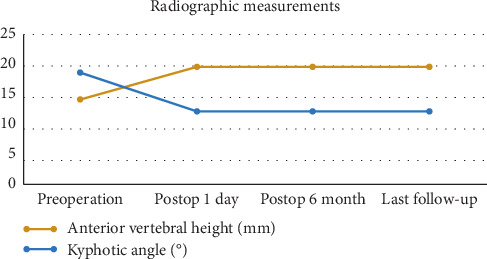
Line charts for radiographic measurements (*n* = 29).

All the 29 patients with Kümmell's disease underwent the PVP procedure successfully. The follow-up period of all patients ranged from 16 to 24 months (mean 19.6 ± 2.2 months). The mean operation time was 35 ± 12 min. All patients were able to walk/ambulate with a thoracolumbar brace after 12 to 24 hours staying in bed postoperatively. The hospital stay was 3.2 ± 0.9 days (range, 2–4 days). The BP-VAS score was reduced from 8.37 ± 1.06 preoperatively to 2.45 ± 1.21, 2.26 ± 1.13 2.13 ± 0.97, 2.16 ± 1.01 and 2.12 ± 0.92 at 1 day, 1 month, 6 months, 12 months, and last follow-up, respectively, after PVP. The ODI score was also decreased from 73.60 ± 10.23 preoperatively to 26.33 ± 5.17, 22.87 ± 4.62, 20.15 ± 3.93, 19.82 ± 3.61, and 20.07 ± 3.24 at 1 day, 1 month, 6 months, 12 months, and last follow-up, respectively, after PVP. Significantly statistical differences were observed in both BP-VAS and ODI scores at each time point of follow-up when compared with the preoperative condition (*P* < 0.05, Table 2, Figures 3 and 4). Besides, statistically significant improvement in radiographic measurements such as kyphotic angle and the height of the anterior border of the involved vertebral body between the preoperative and postoperative assessments were also observed (*P* < 0.05). The detailed data of the radiographic outcomes are summarized in Table 3 and Figure 5. Cement leakage was assessed by postoperative immediate X-rays and CT scans. The leakage of bone cement occurred in 7 of the 29 cases (24.1%), including 3 paravertebral soft tissues, 4 intradiscal, and no leakage was found in the spinal canal. Besides, no surgical complications such as neurological deficit, pulmonary embolism, thermal injury, infection, delayed displacement of bone cement, or adjacent vertebral fracture were observed. And all patients presented no exacerbation for internal medical comorbidities.

## 4. Discussion

In clinical practice, Kümmell's disease is diagnosed as delayed vertebral collapse, dynamic instability, and progressive kyphosis with prolonged back pain and/or neurological deficit after an asymptomatic or mild period [1, 7, 15]. In recent years, with the increasing number of patients with osteoporosis and the improvement of medical imaging technology, more and more cases of Kümmell's disease have been identified. The intravertebral vacuum sign is found on the X-ray and CT scans, and the vacuum cleft is accentuated on the extension views and may shrink on the flexion views. A diffuse low-intensity area or confined high-intensity in the fractured vertebrae is detected on T2-weighted MR images, which depends on gas or fluid-filled cavity. In previous literature, Kümmell's disease was also described as intravertebral vacuum cleft, intravertebral vacuum sign, vertebral osteonecrosis, delayed vertebral collapse, or vertebral pseudarthrosis. Up to now, the pathogenesis is still controversial, and two theories are favored in the literature. Based on the first theory, intravertebral vacuum cleft was caused by vertebral osteonecrosis [11, 16], while the second considered that intravertebral vacuum cleft signifies vertebral nonunion and pseudarthrosis [17, 18]. Perhaps, several factors interact with each other and eventually lead to Kümmell's disease.

The disease does not heal over time and is a sustained source of back pain and disability. These patients are refractory to conservative management such as narcotic analgesics, bed rest, and brace fixation. Surgical intervention may be required to eliminate abnormal activities, relieve the back pain, and to prevent further vertebral collapse, hence preventing kyphotic deformity. Based on the imaging manifestations and clinical features, Li et al. [19] classified Kümmell's disease as 3 types. All patients in our study were type I and II without neurologic deficit. For patients with neurologically intact Kümmell's disease, open surgery is not a preferred choice, especially in elders or osteoporotic patients, and minimally invasive surgery such as PVP has acquired good clinical outcomes [10, 12–14]. The ultimate treatment plan should be individualized.

In the current study, the results showed that the anchoring technique of PVP can significantly relieve the back pain, recover the height of the affected vertebral, and correct the kyphosis deformity. Kümmell's disease often has the characteristic of pseudoarthrosis. The manipulative reduction in the hyperextension position which was introduced by Li et al. [20] was useful in the recovery of the collapsed vertebral height (Figures 1(e) and 2(h)). Zhang et al. considered that the necessity and effectiveness of balloon usage in percutaneous kyphoplasty (PKP) are greatly reduced after acquiring a satisfactory hyperextension position [14]. Because of the liquid or gaseous cavity in the vertebra, the bone cement can flow without resistance during the process of unilateral injection. The symmetric distribution of the bone cement was verified by the fluoroscopic imaging (Figure 1(g)). The anchoring technique in PVP was presented as follows. In our study, the bone cement was injected successively in two states. First, high-viscosity PMMA was injected under fluoroscopy during the early stage of the dough period to plug the gap. Second, the low-viscosity PMMA was prepared at the early stage of the wire drawing period and injected cautiously and slowly. While adhering to the surface of the previous mass of bone cement, the low-viscosity PMMA quickly diffused into the cancellous bone. Besides, appropriate tailing in the pedicle was performed before the end. Then, the low-viscosity PMMA was firmly fixed in the vertebral body like a ship anchor to prevent the slide of the high-viscosity mass of bone cement (Figure 1(h)). The process of injection was tightly monitored under fluoroscopy. After staged injection in the anchoring technique of PVP, our patients all acquired partial or complete back pain relief, which was attributed to the stabilization of the spinal column. The results showed that back pain VAS scores had significantly decreased after surgery during the follow-up period. Besides, pain relief improved the quality of life, as evident from the significant statistical decrease of postoperative ODI scores. As well known, patient-reported VAS and ODI scores are of crucial importance when evaluating the clinical efficacy of surgical intervention [21]. In addition, radiographic outcomes further confirmed the efficacy of anchoring technique in PVP. Results indicated that there was a certain postoperative correction of the kyphotic angle and restoration of the vertebral height, which were not statistically lost during the follow-up.

Previous studies reported a high rate of cement leakage in Kümmell's disease treated by PVP, which ranged from 55 to 75% [13, 22, 23]. But Zhang et al. [14] reported that the rate of cement leakage was 36.4%, and Park et al. reported that the leakage rate was 26.3% [12]. In the current study, the leakage of bone cement occurred in 7 of 29 cases (24.1%), including 3 paravertebral soft tissues, 4 intradiscal, and no leakage was found in the spinal canal. Peh et al. [13] considered that the higher rate may be attributed to the intravertebral cleft, as leakage into paravertebral soft tissues and intradiscal almost occurred at the location of the crack. The leakage into disc may increase the risk of adjacent vertebral fracture because of the increased mechanical pressure resulting from the bone cement [7]. But in our study, no adjacent vertebral fracture was observed during the whole follow-up period. Besides, delayed displacement of bone cement is a rare but serious complication of Kümmell's disease following PVP [24]. But no delayed cement displacement occurred in our study, which may be contributed by applying the anchoring technique in PVP or attributed to the integrity of the anterior structure. No other surgical complications such as the neurological deficit, pulmonary embolism, thermal injury, or infection were found.

Our study has certain limitations. First, it was a single-centre retrospective study with small sample size. Second, the duration of follow-up varied which might exert an effect on our results. Finally, as the goal of PVP was mainly to strengthen the collapsed vertebrae without aiming for neurological decompression [25], this minimally invasive technique was not a preferred option for the patients with neurological deficits.

## 5. Conclusion

The anchoring technique in unilateral PVP had achieved satisfactory clinical and radiological outcomes, and we considered that this minimally invasive procedure could provide an effective and safe alternative for the treatment of patients with neurologically intact Kümmell's disease. But a prospective study with a larger sample and longer-term follow-up is needed to draw a more convincing conclusion.

## Figures and Tables

**Table 1:** Baseline characteristics of the patients.

Characteristics of patients *(n* *=* *29)*	
Male/female	17/12
Age (years)	73.8 (range: 67–81)
Duration of symptoms (months)	4.3 ± 1.2 (range: 2–7)

*Level*	
T7	*n* = 2 (6.9%)
T10	*n* = 2 (6.9%)
T11	*n* = 4 (13.8%)
T12	*n* = 9 (31.0%)
L1	*n* = 7 (24.1%)
L2	*n* = 3 (10.3%)
L3	*n* = 2 (6.9%)
Duration of follow-up (months)	19.6 ± 2.2 (range: 16–24)

*Note*. T: thoracic vertebrae; L: lumbar vertebrae.

**Table 2:** The back pain VAS and ODI score preoperatively and at each time point postoperatively (*n* = 29, mean ± standard deviation).

	Preoperation	Postop 1 day	Postop 1 month	Postop 6 months	Postop 12 month	Last follow-up
BP-VAS	8.37 ± 1.06	2.45 ± 1.21^*∗*^	2.26 ± 1.13^*∗*^	2.13 ± 0.97^*∗*^	2.16 ± 1.01^*∗*^	2.12 ± 0.92^*∗*^
ODI	73.60 ± 10.23	26.33 ± 5.17^*∗*^	22.87 ± 4.62^*∗*^	20.15 ± 3.93^*∗*^	19.82 ± 3.61^*∗*^	20.07 ± 3.24^*∗*^

*Note.* ^*∗*^ *P* < 0.05, score at each time point postoperatively vs. preoperative score. Postop: postoperative; BP-VAS: back pain VAS. Last follow-up occurred at 19.6 months on average, ranging from 16 to 24 months.

**Table 3:** Radiographic measurements of anterior vertebral height and kyphotic angle (*n* = 29, mean ± standard deviation).

	Preoperation	Postop 1 day	Postop 6 months	Last follow-up
Anterior vertebral height (mm)	14.17 ± 2.32	19.53 ± 2.77^*∗*^	19.48 ± 2.83^*∗*^	19.36 ± 2.79^*∗*^
Kyphotic angle (°)	18.55 ± 4.47	12.07 ± 3.23^*∗*^	12.16 ± 3.51^*∗*^	12.33 ± 3.39^*∗*^

*Note.* ^*∗*^ *P* < 0.05, data at each time point postoperatively vs. preoperative data; the kyphotic angle was marked with black lines in Figure 2(a); last follow-up occurred at 19.6 months on average, ranging from 16 to 24 months.

## Data Availability

The data used to support the findings of this study are available from the corresponding author upon request.
